# Mitigating the stress response to improve outcomes for older patients undergoing emergency surgery with the addition of beta-adrenergic blockade

**DOI:** 10.1007/s00068-021-01647-7

**Published:** 2021-04-13

**Authors:** Shahin Mohseni, Bellal Joseph, Carol Jane Peden

**Affiliations:** 1 Division of Trauma and Emergency Surgery, Department of Surgery, Orebro University Hospital, 701 85 Orebro, Sweden; 2 Faculty of Medicine and Health, School of Health and Medical Sciences, Department of Surgery, Orebro University, 702 81 Orebro, Sweden; 3 3Division of Trauma, Critical Care, Emergency Surgery, and Burns, Department of Surgery, University of Arizona College of Medicine, Tucson, Arizona USA; 4 University of Southern California, Los Angeles, USA; 5 Unniversity of Pennsylvania, Philadelphia, USA

**Keywords:** Emergency surgery, Surgical stress, Elderly, Geriatric, Beta-blockers

## Abstract

As population age, healthcare systems and providers are likely to experience a substantial increase in the proportion of elderly patients requiring emergency surgery. Emergency surgery, compared with planned surgery, is strongly associated with increased risks of adverse postoperative outcomes due to the short time available for diagnosis, optimization, and intervention in patients presenting with physiological derangement. These patient populations, who are often frail and burdened with a variety of co-morbidities, have lower reserves to deal with the stress of the acute condition and the required emergency surgical intervention. In this review article, we discuss topical areas where mitigation of the physiological stress posed by the acute condition and asociated surgical intervention may be feasible. We consider the impact of the adrenergic response and use of beta blockers for these high-risk patients and discuss common risk factors such as frailty and delirium. A proactive multidisciplinary approach to peri-operative care aimed at mitigation of the stress response and proactive management of common conditions in the older emergency surgical patient could yield more favorable outcomes.

## Background

Around the world populations are ageing rapidly. This ageing, while a source for celebration of improved population health interventions and healthcare, also presents a worldwide challenge to all health care systems [1–3]. In 2019, there were 703 million people aged 65 or over worldwide. The percentage of patients termed the “oldest old”, those over 80 years of age, is also increasing in all countries and currently constitutes about 7% of the world’s 65 years and over population but is projected to triple between 2005 and 2030 [4]. Older patients will need surgery, and while some of the impact of ageing can be mitigated by careful preparation and planning for surgery, that is not the case when urgent or emergent care is required. It is inevitable that growing numbers of older patients will require acute surgical care [5], and there is a need to consider how best to minimize the stress of unplanned surgery on this at-risk population.

In contrast to elective surgery, patients needing emergency surgery typically present with limited time for pre-operative optimization and in a state of heightened physiological stress. This stress is in direct relation to the patient’s disease acuity and is mediated by a surge in catecholamine release that is further exacerbated by the operative intervention itself [6]. Further, and as a consequence of advanced age [6–8], an increased burden of medical comorbidities [9], frailty [10–13], polypharmacy and diminishing physiological reserves [14], older patients undergoing emergency surgery represent a unique group with an increased risk of mortality and a disproportionate burden of complications [5, 8, 14–16].

Improving outcomes for geriatric patients undergoing emergency surgery remains a challenging task that necessitates comprehensive evaluation in parallel with a multidisciplinary approach to care. Understanding the various risk factors for, and the associated systemic effects of the excessive hyperadrenergic state, is of paramount importance to guide targeted interventions. The aim of this review is to summarize and critically appraise current evidence on stress in geriatric patients undergoing emergency surgery and to consider ways to mitigate its toxic consequences in an effort to improve outcomes.

## Emergency surgery in the elderly—in this paper, we chose to focus on emergency intra-abdomial and hip fracture surgery, both common procedures in the elderly with high risk of perioperative adverse outcomes

Around the world, emergency surgical procedures are associated with a much poorer outcome than elective procedures [17, 18]. Older age, increasing comorbidity and non-elective surgery are independent predictors of death and complications in non-cardiac and non-neurosurgical patients [17, 18]. One large cohort study from the United Kingdom (UK) defined high-risk surgical procedures as those with a 30 days mortality greater than 5%. The study found that this group accounted for only 12.5% of surgical in-patient procedures but represented 80% of deaths; two of the top five surgeries with the highest mortality rates were emergency bowel procedures in older patients and surgical repair of hip fracture in patients over 69 years of age [19]. Not only do these procedures have a high mortality, but they constitute together, a large volume of emergency surgical procedures. In the United States, emergency general surgery makes up 8–26% of all hospital admissions, and represents 11% of all surgical admissions [20–22]. However, it represents an excess of 50% of all surgical mortalities [23]. In Sweden, the 30-day mortality after surgery for colon cancer, considered a disease of the elderly with the mean age of patients being 70 years, is 7.5% for emergency surgery compared with 1.7% for elective surgery [24]. This finding may be explained to some extent, by the widespread national implementation of multidisciplinary cancer programs, centralization of care, and implementation of pathways such as Enhanced Recovery After Surgery (ERAS) guidelines [25], which promote timely diagnosis, pre-operative optimization and protocolized perioperative care in elective settings. In contrast, emergency surgical patients present with physiological derangement out of hours when some healthcare systems may not be able to reliably provide the same “highest quality of care” to that which could be delivered during normal working hours. Issues include no senior clinicians in-house and radiology limitations [26, 27]. The surgical team must, therefore, deal with system issues, patient issues (deranged physiology and the impact of comorbidity and ageing) as well as the surgical procedure itself. Timely assessment and diagnosis, pre-operative optimization, and early intervention by the best team available may be difficult and may require system redesign to be achieved reliably [28].

The other very common high risk presentation for older surgical patients in addition to emergency general surgery are those with a fractured neck of femur, i.e. hip fracture. Again, internationally this procedure carries a high burden not only for patients and families but also for health care systems. Volumes are high with approximately 2.2 million procedures performed annually worldwide, and are expected to rise to 4.5M by 2050 [29]. Although the physiological disturbance may not be as great as that seen in patients with intra-abdominal pathology, patients with a fractured hip suffer pain and a physiological stress response including inflammation, catabolism and disordered coagulation. Mortality and morbidity are high after hip fracture surgery and patients that do survive the initial insult often have significant functional decline and increased postoperative mortality risk [30]. While some risk factors such as age and comorbidities cannot be modified, other components of care such as multidisciplinary management, time to the operating room, and early optimization have been shown to improve outcomes [30, 31].

## Beyond age: assessment of other risk factors that impact the stress response and adverse outcomes

Age has long been considered an independent risk factor for adverse outcomes and mortality after surgery [7, 8], even in low-risk general surgical procedures such as appendectomy [32, 33]. The early post-operative mortality risk increases substantially for every decade after 60 years of age in patients subjected to high-risk surgical procedures such as emergency laparotomy, culminating in a dismal predicted survival of less than 10% in patients over the age of 90 years [8]. Aside from the age factor, frailty and cognitive impairment are two distinct, but common conditions in geriatric patients who present with a surgical emergency. Aging has also profound effect on the systemic stress response by blunting the hypothalamic pituary axis feedback or decresing the blood concentration of other hormonal mediators, such as glucocorticoids [34, 35].

### Frailty

Frailty is a clinical syndrome of decreased functioning of multiple organ systems and decreased resistance to stressors, with a reported incidence of between 23% to 52% in the geriatric population [11, 12, 36, 37]. It is a multidimensional phenotype that measures the state of depleted physiological reserves that are essential for recovery to the pre-injury functional level [10, 13]. There is a spectrum for frailty, which includes the concept of pre-frailty a potentially reversible condition before onset of established frailty, and there is now a great deal of evidence showing a strong association between increasing degree of frailty (including pre-frailty) and higher postoperative mortality [38, 39]. The range of frailty can be assessed with common scales such as the Clinical Frailty Scale which ranks patients from very fit to terminally ill, or more complex scales such as the NSQIP derived Risk Analysis Index-Administrative which is a continuous variable with a range from 0 to 81, with higher scores indicating greater frailty [38]. The use of a frailty index as an assessment tool for identifying high-risk surgical patients carries the advantage of accounting for a patient’s physiological, cognitive, psychological, and social deficits into account. The proposed comprehensive assessment has been deemed by multiple studies to be far superior to a patient’s chronological age in predicting worse outcomes after surgery [11, 12, 36, 37]. Interestingly, the association between frailty and mortality remains after accounting for case-mix with different physiological stress responses caused by different surgical procedures among several non-cardiac surgical specialities, with significant increase in mortality in frailer patients [40]. Additionally, the surgical stress in frail geriatric patients is additive, and thereby amplified in cases of postoperative complications. This highlights the concept of not only complication prevention, which is not always possible, but also complication management. The ability to respond to patients’ postoperative complications, and, therefore, prevent death following such complications, may present high-yield targets for quality improvement [41, 42].

### Cognitive impairment

Hewit et al. reported a 66.9% rate of cognitive impairment in a prospective observational study evaluating geriatric emergency surgery patients [43]. Cognitive impairment is an independent risk factor associated with a heightened risk of postoperative complications, including sepsis, delirium, and the need for reoperation [44]. The importance of identifying patients with some form of cognitive impairment starts early on with screening, and then deciding on a management plan. Patients must have the capacity to weigh the risks and benefits of the treatment options proposed even in the emergency setting, in compliance with the informed consent process [45].

Delirium, is an acute state of inattention and confusion and is a frequent complication encountered in the geriatric population postoperatively, with reported rates ranging from 7% to 65% [15, 46–49]. Multiple intrinsic and extrinsic factors come into play when determining a patient’s delirium risk. The intrinsic risk factors correspond to the depleted physiological reserve in older adult patients, which puts them at a heightened risk for postoperative decompensation on multiple organ levels, including their cognitive status. The extrinsic risk factors are those related to the perioperative care and the operative intervention itself [50]. There is debate over the pathophysiology underlying the development of delirium but suggested causes include a sympathetic nervous system stress response to surgery and perioperative neuroinflammation [50].

Delirium is associated with serious adverse health outcomes, more pronounced in the emergency surgery setting. Geriatric patients with a hospital stay complicated by delirium are more likely to experience ICU admission, unplanned intubation, and a prolonged hospital stay, with increased mortality rates [51]. This underlines a major stress reaction that can be potentially mitigated or avoided if timely and effectively recognized. Older age, frailty, polypharmacy, comorbidity, and the use of hypnotics and sedatives are risk factors for the development of delirium in surgical patients [51, 52]. Comprehensive initial assessment of geriatric patients admitted for emergency surgery allows for identifying patients at risk, and close monitoring. Sepsis and pain are well recognized causes of delirium which may be present in the older patient presenting for emergency surgery. Key management components are treatment of the underlying condition with surgery as necessary, and appropriate resuscitation and pain management. If the patient is unable to provide informed consent for surgery the appropriate local legal processes shoul be followed [53]. As stated above, patients who experience delirium have an increased incidence of complications and mortality, and if they survive there is an association with an increased risk of neurocognitive disorder and dementia. These are significant risks and so should be discussed as part of the informed consent process. The sooner delirium is detected through proactive screening, the sooner appropriate mitigating action can be taken. There are a number of recent guidelines on best practices to reduce the incidence of delirium in older surgical patients [54–57]. Additionally, the judicious consultation of geriatric services as part of the multidisciplinary care team can aid in modifying the underlying risk factors in an effort to minimize postoperative stressors [16].

Overall, preoperative assessment of frailty and cognitive impairment can assist in an individual goal-directed surgical care, and, therefore, need to be incorporated into the management protocols of older patients requiring emergency surgery.

## The hyperadrenergic state in surgical patients

The hypothalamic activation of the sympathetic autonomic nervous system resulting from surgical trauma, increases the secretion of catecholamines from the adrenal medulla and the release of norepinephrine from presynaptic nerve terminals. The increase in sympathetic activity has a profound effect on the cardiovascular system, and also affects the function of several visceral organs including liver, pancreas and kidneys, as well as modulating the proinflammatory response [6]. Although a natural response, the catecholamine surge, triggered by acute conditions such as sepsis and trauma, has several negative implications if sustained and not mitigated. These negative implications include cardiovascular overload, insulin resistance, increased inflammatory reaction, and hypercoagulability; all of which potentially worsen postoperative outcomes [6, 58].

Beta-receptor activation has been shown to play a major role in the immunomodulation at times of physiological stress [59–62], and high levels of circulating catecholamines after traumatic injury have been independently associated with an increased mortality risk [63]. The increased risk of cardiovascular insults due to increased catecholamine levels in the peri- and post-operative period after major surgery has also been known for decades [6, 58, 64], with a diagnosis of cardiac condition prior to surgery being an independent risk factor for peri-operative myocardial infarction, and subsequent increased risk of mortality [65].

## Minimizing the stressors of surgery

### Prompt diagnosis

Acute abdominal pain is one of the most common symptoms in elderly patients presenting to emergency departments in the United States [66]. The evaluation of abdominal pain in this patient population is especially challenging since there is a higher prevalence of comorbidities and polypharmacy, both of which can attribute to unreliable vital signs and laboratory values. At the same time, a likelihood of a life-threating pathology must always be taken into consideration during diagnosis, and investigation should be undertaken with a sense of urgency [65, 67–69].

### Imaging

The importance of early and liberal imaging in the elderly population with undifferentiated abdominal pain can not be overstated, and is strongly emphasized in the emergency medicine literature [70]. An immediate interpretation of the images should be carried out by a radiologist for guidance in diagnosis and to minimize the delay to definitive care. The time spent for obtaining radiology, most notably CT scan with intravenous contrast, and the review by a radiologist can potentially be a time limiting factor for delivering definitive surgical care, and therefore needs to be addressed in clinical practice guidelines and pathways [71]. One large study showed that greater institutional use and availability of computerized tomography for emergency general surgical admissions, was an independent predictor of lower mortality [72].

### Time to surgery

In geriatric patients, acute conditions represent a physiological strain that may exacerbate the underlying condition and deplete the reserves needed for rehabilitation after necessary surgical interventions have been undertaken. In many emergency surgery cases, source control for conditions such as sepsis due to intra-abdominal hollow viscus perforation or removal of the organ (gallbladder, appendix) causing the systemic inflammatory reaction are the only options to address the physiological stress, with delays to surgical intervention posing an increased risk of post-operative morbidity and mortality [73, 74]. Several studies investigating the implementation of care bundles for emergency general surgery with a targeted time to surgery within 6 h have consistently found improved outcomes [73, 75, 76].

Multiple small studies and cohort analyses have shown that patients with hip fracture have better outcomes if surgery is carried out without uneccesary delay after the injury, and that surgery should be delayed only if the benefits of additional medical treatment outweigh the risks of delaying surgery. Delphi consensus guidelines state that surgery should occur within 48 h of the injury [29]. A recent multi-center international randomised controlled trial “HIP ATTACK” compared outcomes of patients in whom the median time to surgery was 6 h from the time of fracture, compared with those in whom the median time to surgery was 24 h. There was no difference in mortality or the incidence of major complications at 90 days between the two groups, but the accelerated patient group had a reduced risk of delirium, urinary tract infection, and moderate-to-severe pain, faster mobilization, standing, weight bearing, and hospital discharge [31].

### Surgical approach

A minimally invasive approach is always preferred in situations where patient characteristics, access to equipment, and surgeon skill allow, in an effort to minimize the surgical trauma [77]. For many emergency surgical diseases, such as appendicitis and cholecystitis, the laparoscopic approach is the gold standard approach today [78]. Additionally, the pendulum is moving towards the minimally invasive approach in the more technically challenging acute surgical cases such as bowel obstruction and perforation in an effort to reduce the surgical trauma and enhance the postoperative rehabilitation [79, 80].

### Pain management

The negative effect of pain on healing and rehabilitation after surgery is undisputable. In the elderly, it is even more important to use a multimodal pain management approach to decrease the reliance on perioperative opiods to reduce the risk of falls, delirium, promote earlier bowel movement, and with the goal to facilitatate mobilization and recovery. However, high-quality data on pain management, especially in geriatric patients subjected to major general surgery (i.e. laparotomy) is scarce. Nevertheless, the recommendation for multimodal pain management strategies from general elective surgery, with a goal to reduce opioids in favor of non-opioid analgesics and techniques for faster recovery is recommended in all geriatric surgical cases [81–84]. In hip fracture surgery, the use of regional anaesthesia prior to surgery has been shown to be effective in reducing the incidence of pneumonia and time to first mobilization.

### Nutrition

Malnutrition is common in elderly patients undergoing emergency surgery and is associated with worse postoperative outcomes [85, 86]. Unimposed physiological stress by acute conditions and subsequent surgical interventions may lead to a relative insulin resistance with elevated circulating glucose levels, which can have negative effects on patient outcomes [87]. A balanced approach with replacing the needed caloric intake required for rehabilitation while considering the risk of hyperglycemia is of importance [88]. Early enteral nutrition, even after gastroentistinal emergency surgery, is recommended [89].

### The role of beta blockers in mitigation of the stress response

Preoperative use of beta-blockers in non-cardiac surgery has been a matter of controversy over the past few decades. As more data have emerged, the recommendation from the American College of Cardiology/American Heart Association (ACC/AHA) changed from advocacy of the initiation of perioperative beta -blocker to a Class IIa recommendation (i.e. it is reasonable) in patients in whom “preoperative assessment identifies untreated hypertension, known coronary disease, or major risk factors for coronary disease” [90, 91].

The most recent guidelines (from 2014) advocate for continuing beta blockers in patients on regular preoperative treatment, and considering initiation in intermediate to high-risk patients [≥ 2 clinical risk factors, ASA class ≥ III, and ≥ 3 factors from the revised cardiac risk index (RCRI)] [92, 93]. Interestingly, when patients are stratified by the revised cardiac risk index, there is a clear association with the beneficial effects of perioperative beta-blocker treatment with higher RCRI values (RCRI score 2–4), and potential harm with RCRI values of 0–1 [94, 95]. These recommendations make the use of preoperative beta blocker for cardiovascular protection more reasonable in the geriatric population due to higher prevalence of both diagnosed and undiagnosed cardiovascular conditions. Further, older patients are to a greater extent frail, and caridiopulmonary complications are a significant contributer to mortality in patinets with perioperative frailty [38].

Sepsis and septic shock are common in patients admitted for emergency general surgery, and have been strongly associated with mortality [96]. Older patients are particularly vulnerable, a study of 360,000 patients in the NSQIP database found that the presence of any comorbidity increased the risk of sepsis and septic shock by sixfold, and increased the 30-day mortality rate by 22-fold [97]. It has been postulated that the increased risk for cardiac dysfunction, hyperglycemia, and hypercoagulability seen in septic patients is, to a large extent, is a consequence of the hyperadrenergic state [98–100]. Several studies have found improved tissue perfusion, hemodynamics, fluid requirements, and cardiac function in critically ill septic patients receiving beta-blockers [101–104]. The randomized controlled trial conducted by Morelli and colleagues, allocating septic patients to esmolol treatment or not, found a 61% reduction in 28-day overall mortality in treated patients (adjusted hazard ratio 0.39, 95% CI 0.26–0.59) [105]. Interestingly, although Singer and colleagues detected a survival benefit (AOR 0.69, 95% CI 0.62–0.77) in patients on preadmission beta-blockers, they found a more profound effect in the non-cardiac selective beta-blocker cohort, which would speak to the pleiotropic effects of beta-blockers [100]. The authors speculated that this finding could be due to the downregulation of the catecholamine surge mediated inflammatory cascade and increased cytokine release, more than the cardioprotective effect of beta-blockers [100, 106–108].

In trauma patients, beta-blocker administration has shown a down-regulation of proinflammatory factors such as interleukin-6 and tumour necrosis factor-*α* [109, 110]. Catecholamine surge has been shown to be very pronounced in patients with severe traumatic brain injury (TBI) [111–113], where the hyperadrenergic state has strongly been linked to immunomodulation within the brain and systemically, worsening both the secondary brain injury and leading to extracranial organ dysfunctions [61, 62, 114–116]. In animal TBI models beta-blocker receptor inhibition has shown better intracerebral perfusion, oxygynation, glucose metabolism, decreased inflammatory response, and better motor performance [117, 118]. More importantly, the TBI induced cathecolamine surge has been associated with hypermetabolism, myocardial necrosis, cardiac arrythmias, pulmonary hypertension and non-cardiogenic pulmonary edema, contributing to an increased risk of extracranial morbidity and mortality [115, 119–122]. Several studies have attributed the strong association between increased survival after severe TBI and post-trauma beta-blocker exposure to the down-regulation of the toxic effects of the hyperadrenergic state on the extracranial organs [123–127]. At the moment, beta blocker is part of the Lund-Concept Protocol for severe TBI management [128], and is conditionally recommended by The Eastern Association for the Surgery of Trauma (EAST) in these instances to mitigate the adverse impact of the trauma induced stress on both the injured brain tissue and extracranial organs [127].

Surgery by itself is a trauma and more data is emerging that beta-blockade in the context of non-cardiac general surgery is associated with better post-operative survival [24, 64, 94, 129–132]. In a large retrospective analysis from the prospectively maintained Swedish Colorectal Cancer Registry, including only patients undergoing surgery for rectal cancer, a significant decrease in post-operative complications and decreased risk of 30-day mortality was detected in patients with an active beta-blocker prescription prior to the date of surgery [129]. The same results were obtained using the same database investigating the association between pre-operative beta blocker use and mortality after surgery for elective colon cancer [24]. For emergency unplanned cases of colon cancer surgery, despite the fact that the beta-blocked patients were older and less fit for surgery based on their preoperative ASA classification, and burdened by higher rates of comorobidities, they had a decreased risk of postoperative mortality [crude 30-day mortality: 8.6% vs. 3.1%; adjused incidence rate ratio (IRR) for 30-day mortality: 0.31, 95% CI 0.19–0.47; *p* < 0.001] [133]. Interestingly, there was a decreased risk of cardiovascular (adj IRR 0.33, 95% CI 0.12–0.92, *p* = 0.34) and septic (adj IRR 0.2, 95% 0.05–0.84, *p* = 0.029) deaths in the beta-blocker cohort [133].

In recently published studies examining patients with isolated hip fracture undergoing surgical repair, an association between pre-admission and continuous in hospital beta-blocker therapy and improved survival was noted [134]. Using the Swedish National Quality Register for hip fractures (Rikshöft), including a total of 126,934 geraitric patients (over 65 years of age) of hip fractures with a mean age of 84 years, a 70% risk reduction for 30-day mortality (Ajd. IRR 0.31, 95% CI 0.29–0.32, *p* < 0.001) in patients with pre-admission ongoing regular beta-blokcer therapy could be detected [135]. This is of particular significance since the majority of patients with hip fractures are older adults with a mean age of 80 years with several comorbidities, with the cause of post-operative death overwhelmingly attributed to cardiopulmonary and/or multiorgan failure [135]. Compared with emergency general surgery cases, surgical hip fracture repair is usually “a more straightforward and less complex surgery”. Therefore, the proposed theory that the stress caused by the trauma and surgical intervention leads to a hyperadrenergic state causing remote organ damage and ultimately worse outcomes is not far-fetched.

However, it must be stressed that high-quality data for the perioperative use of beta blockers is still needed. The POISE trial, the largest randomized control study of perioperative beta blockade to date, detected an increased mortality risk with preoperative beta-blocker administration despite a reduced risk of cardiac adverse events in the perioperative period [136]. There were several limitations to this study including the case mix which was sub-divided into vascular, orthopedic, and intra-abdominal procedures all of which have different patient risk profiles and include a wide variety of procedures each with different postoperative morbidity and mortality risks. Another major critique was the dosage of extended-release metoprolol 100 mg h before surgery to beta-blocker naïve patients, with repeated dose 6 h postoperatively. These high dosages could account for the higher risk of stroke, hypotension and bradycardia, seen in the POISE trial [136], which have not been detected in several other observational studies where lower doses of beta-blocker were used [137, 138].

Despite promising associations noticed between beta-blocker exposure and decrease in adverse outcomes, the role of beta-blockade in elderly patients subjected to emergency surgical procedure needs further investigation including the timing of initiation, type of beta-blocker used, dosage, and length of treatment.

## Improving the system and delivery of multidisciplinary care

The need to address the high morbidity and mortality of common emergency surgical procedures has led to the emergence of care bundles and defined care pathways in an attempt to rapidly and reliably deliver evidenced based care in emergency situations. In emergency general surgery, and specifically one of the highest risk procedures emergency laparotomy, a number of studies have shown benefit, with reduced mortality, length of stay, reduced complications and increase delivery of evidence based processes [75, 76, 139]. These successful studies kept the number of interventions small, and targeted them at rapid correction of deranged physiology. For example Aggarwal and colleagues implemented a six component care bundle across 9247 patients undergoing emergency laparotomy with improved outcomes compared with a baseline group [139]. All the processes were directed at rapid mitigation of the stress response and physiological derangement. The components were: immediate screening for abnormal physiology, SIRS and sepsis including an arterial lactate; immediate management of deranged physiology; urgent radiology and diagnosis; urgent source control and surgery; goal directed fluid therapy; early involvement and presence of senior anesthesiologists and surgeon; and postoperative ICU for all [139].

For patients with hip fractures, interventions to standardize patient care have improved outcomes. One of the largest hip fracture database in the world is in the UK, has since its inception in 2007 shown an almost year on year decrease in mortality with 2018 data showing a 6.1% 30 days mortality across 66,000 patients. Improvements in outcomes have been driven by a focus on multidisciplinary management with involvement of geriatricians, surgery within 36 h, routine screening for delirium, and early mobilization in the perioperative phase of care [140]. In the UK, this work has been supported by a payment system to meet quality standards. A recent systematic review found that co-management of patients with geriatric physicians improved outcomes with the potential to markedly reduce the costs of care [141].

To reliably deliver care to high-risk older surgical patients, anesthesia and surgical teams must work together with emergency physicians, hospitalists and geriatricians to develop pathways of care which prioritize correction and minimization of the response to the underlying insult, minimize delays and promote early mobilization and feeding in patients who may have little reserve. A multimodal team approach to the whole perioperative journey is used in Enhanced Recovery after Surgery (ERAS) protocols. Using an enhanced recovery approach has been shown to be effective in improving outcomes for elderly emergency surgical patients [27, 75, 76, 139, 142].

There is now much greater awareness amongst national organizations that care for older surgical patients. The Society of Perioperative Assessment and Quality Improvement (SPAQI) has recommended initiation of Geriatric Surgery Verification Program to meet aspects of the perioperative care of geriatric patients [143]. This verification program entails hospitals to adhere to standard measures of care such as routine screening for common geriatric complexities, engaging patients and their families in the decision-making process, and ensuring delivered care is in line with patient goals [143]. The American College of Surgeons has launched a geriatric verification program for hospitals and the American Geriatrics Society and the American College of Surgeons have issued joint guidelines on optimal care of the geriatric surgical patient [144]. Finally, with the linkage between higher hospital and surgeon volume of emergency surgical cases and improved outcomes, specially in geriatrics, designated hospitals should be considered for this patient population in the future [145, 146].

## Conclusion

Compared with elective surgery, patient optimization in emergency surgery is limited preoperatively due to circumstances. Optimal surgical care must include management of the underlying acute physiological state, the consideration of issues affecting older patients including co-morbidities, frailty and cognitive dysfunction, and system issues related to delivering complex care at all hours. Expeditious recognition, assessment, diagnosis, resuscitation, and intervention for surgical emergencies along with decreasing the amplitude of the catecholamine surge to mitigate its toxic systemic effects are essential for better postoperative outcomes in the geriatric patient.
